# Virtual Reality-Based Exercise with Exergames as Medicine in Different Contexts: A Short Review

**DOI:** 10.2174/1745017901915010015

**Published:** 2019-01-31

**Authors:** Marcos Túlio Silva Costa, Lanna Pinheiro Vieira, Elizabete de Oliveira Barbosa, Luciana Mendes Oliveira, Pauline Maillot, César Augusto Otero Vaghetti, Mauro Giovani Carta, Sérgio Machado, Valeska Gatica-Rojas, Renato Sobral Monteiro-Junior

**Affiliations:** 1 Department of Internal Medicine, Faculty of Medicine, State University of Montes Claros, Montes Claros, Brazil; 2 Department of Physical Education, State University of Montes Claros, Montes Claros, Brazil; 3 Departament of Neurology, Faculty of Medicine, Universidade Federal Fluminense, Niterói, Rio de Janeiro, Brazil; 4 UFR de Sciences et Techniques des Activités Physiques et Sportives de Paris, Université Paris Descartes, Paris, France; 5 Faculty of Physical Education, Universidade Federal de Pelotas, Pelotas, Rio Grande do Sul, Brazil; 6 Departament of Public Health, University of Cagliari, Cagliari, Italy; 7 Departament of Physical Activity Science, Universidade Salgado de Oliveira, Niterói, Rio de Janeiro, Brazil; 8 Human Motor Control Laboratory, Universidad de Talca, Talca, Chile

**Keywords:** Neurologic diseases, Exergames, Health promotion, Virtual-reality, Cognition, Balance, Motor-function, Neurobiology

## Abstract

There is enough evidence that, nowadays, the sedentary lifestyle is one of the major health problems worldwide, linked to many chronic diseases, including mental comorbidities, systemic hypertension, metabolic dysregulation, and cancer. Although health societies recommend engagement to physical activities, there is an overwhelming number of people remaining sedentary, even knowing the health benefits of regular exercises. One of the main factors that justifies this scenario is the lack of motivation, which is a barrier to people intended to start new habits for health. Considering this previous information, new alternatives for exercises may help people engage in a healthier lifestyle. Technology has contributed to this with devices that allow movements based on virtual reality approaches, including the exergames. These are games available even in commercial devices, as video-games, that allow people to work with different physical components. Furthermore, exergames add cognitive gain through its dual-task characteristic. Moreover, due to the combination of these benefits, they are feasible to acquire, and easy to use. Exergames are not only a potential strategy to reduce sedentary lifestyle but also a good method to improve health gains and rehabilitation in different populations and pathological conditions: older adults, stroke survivors, and Parkinson’s disease. In this review, we aim to demonstrate some conditions that literature supports the intervention with exergames due to its physical and cognitive benefits. Furthermore, at the end of this review, we also explore the neurobiological mechanisms behind virtual-reality based exercises.

## 1 INTRODUCTION

A sedentary lifestyle is the fourth major risk factor for mortality and is related to several chronic diseases [1]. In a study carried out among Thai workers of an oil company, sedentary behavior (*e.g.* sitting) was positively associated with four pathological conditions, accounting for more than 38 million deaths globally (cardiovascular disease, diabetes mellitus, chronic respiratory diseases and cancer), as well as cardiometabolic risk factors, such as hypertension and hyperlipidemia, which are related to more than 10 million annual deaths [2]. It is estimated that one in four white adults in the United States spends approximately 70% of the time sitting (while awake), with the remaining 30% spent time on activities which demand few efforts or no physical exertion [3].

It is recommended that part of the weekly activities should be dedicated to physical exercise (minimum of 150 minutes/week), which promotes positive metabolic changes, improves cardiovascular capacity, musculoskeletal function, and mental health, as well as reducing the risk of premature death [4]. In addition to an important role in promoting health, physical exercise is a relevant agent in disease management. Wang *et al*. [5] found that even exercises with mild to moderate intensity are beneficial in glucose and lipid control, in addition to reducing mortality from cardiovascular diseases.

The American College of Sports Medicine (ACSM) manages a global health initiative called Exercise is Medicine, which encourages doctors and other health professionals to recommend increasing physical activities of daily living and prescribe physical exercise, when appropriate, due to the benefits promoted by the more active lifestyle [6]. However, even knowing the preventive and therapeutic potential of exercise, a large part of the global population remains sedentary (approximately 31%) [7]. Therefore, different strategies, such as the inclusion of more attractive and motivational activities, should be managed to encourage people to become more physically active.

Many people do not feel motivated to engage in healthy habits, including physical exercise [8]. A meta-analysis showed that approximately 21% of people do not intend to start physical activities, while another 36% have this intention, but present difficulties in changing the sedentary behavior [9]. Therefore, motivation would be an important factor to be stimulated in the engagement of one in a more physically active lifestyle. In this context, Exergames, a Virtual Reality-Based Exercise (VRE), may be a good alternative to increase the level of physical activity, since they have a greater component of fun. According to Oh & Yang, exergames are defined as video games that promote (either *via* using or requiring) players' physical movements (exertion) that is generally more than sedentary and includes strength, balance, and flexibility activities. The exergames require digital devices, such as computers or game consoles and their accessories, as balance boards [10].

For the elderly group, for example, a British study of the national population reveals that, overall, the subjects have in general a low level of physical activity. The analysis can be complex since it is variable between different groups according to sex, socio-demographic, clinical and behavioral factors, as a previous sedentary life [11]. Elderly subjects submitted to this type of intervention showed the greatest involvement with the activity through eight elements of entertainment (concentration, challenge, skill development, sense of control, better definition of goals, feedback, immersion, and opportunity for social interaction) [12]. In addition, there is an increase in confidence, which may be related to self-efficacy and, consequently, it is more likely to persist in the activity [12]. Therefore, the use of exergames can be a good alternative for the reduction of the sedentary lifestyle and, consequently, a decrease in the risk of developing chronic-degenerative diseases. In addition, we can see that exergames can be used in different places (hospitals, residences, long-term care institutions for older adults, and schools) and contexts (such as different age groups and different objectives). As examples, the potential sensory feedback provided by the digital interaction, using the Nintendo Wii Balance Board, was explored to promote improvements in postural balance in children with cerebral palsy [13]. There is also the use in motor learning rehabilitation with impaired motor-function children, exploring the mirror neuron system which promotes the ability to learn through imitation [14], and health promotion in adults, being an alternative in stopping the sedentary lifestyle [15]. In conditions such as cerebral palsy in children, it was used for improving balance [16]. In addition, the technologies required are relatively easily accessible through commercial devices (eg Nintendo Wii and X-box Kinect), which allow their acquisition even in low-income populations and their use even in home-approach [17, 18]. Moreover, VRE can increase adherence, sometimes not achieved with traditional exercises, increasing the probability of obtaining the general health benefits (especially physical and mental status) [13, 18].

In view of the aforementioned context, the aim is to make a brief review on the use of VRE in different contexts, such as cognitive and physical rehabilitation of different audiences. In complement, we intend to explore the possible neurobiological mechanisms of this intervention in order to facilitate the understanding of the effects reached by individuals who use this therapy.

## 2 VRE AND ITS EFFECTS ON COGNITIVE AND MOTOR REHABILITATION OF DIFFERENT POPULATIONS

Exergames are dual tasks which stimulate the brain to generate cognitive and motor responses simultaneously, requiring cortical and subcortical circuitry activation [19]. With the advances of new games, especially interactive games, it is possible to improve balance [20], physical function, processing speed and executive functions [21]. Changes in motor function [22] may be related to cortical reorganization in patients with stroke who were treated using such technology [23].

In a study conducted with children with cerebral palsy, during an individualized training program with VRE, specialists considered that the virtual reality could improve cognitive function through the increase of levels in attention and concentration [24]. In addition, a pilot study using six weeks of training in the residence of patients with spastic hemiplegic Cerebral Palsy (CP) showed improvement of upper limb motor function and an increase of manual force, while another also showed improvement in upper-limb function [25, 26].

In the elderly, VRE has shown positive effects on cognitive functions, both acutely and chronically. Recently, two studies [27, 28] conducted by our group showed such effects. The first study showed that a single session of exergames improved semantic memory and executive function of institutionalized elderly. In support to this, our second study has evidenced that 12-16 sessions of exergames were effective to increase short-term memory and mobility of institutionalized older people. In addition, our results showed maintenance of global cognition over time for individuals who were submitted to the virtual reality intervention, while it did not occur for the other subjects. It is possible that such therapy may prevent the risk factors for dementia. Although still in its beginning, exergame studies on elderly subjects are growing, providing utilization in physical and cognitive training, rehabilitation on neurological and mental diseases and sense of wellness [29].

In a meta-analysis of 17 studies, the potential of exergames to promote cognitive gains, both in clinical and non-clinical populations, was shown to be significantly higher compared to inactive control groups and was still moderately superior to groups with isolated physical activities, that is, without the use of exergames. For the evaluation of isolated cognitive components, exergames were still effective in improving executive function, especially in inhibitory control and flexibility, as well as other functions, such as attentional processing and visuospatial skills. Although metanalysis showed that most of the studies were done with elderly patients (mean age = 69 years), it also included a study conducted with adolescents, demonstrating once again the possibility of using it in different age groups [30]

VRE has shown positive effects on stroke survivors, such as improvement of motor function, balance and cognitive functions [18, 31]. Exercise with moderate to vigorous intensity is essential for maintaining and improving the health of these individuals. In this context, Hurkmans *et al*. showed that exercises with Wii Sports Tennis and Boxing (virtual games of the Nintendo Wii console, which simulate sports movements of tennis and boxing, respectively) can be considered of moderate intensity, since the energy cost evaluated, in Metabolic Equivalents (METs), during the exercises was of 3.7 ± 0.6 METs and 4.1 ± 0.7 METs in Tennis and Boxing, respectively [32]. These findings are consistent with the recommendations of the American College of Sports Medicine and American Heart Association [33] for maintaining cardiovascular health and cardiac rehabilitation (moderate intensity of 3-6 METs). Therefore, the practice of VRE could be indicated to prevent cerebrovascular diseases and, possibly, to promote other health benefits of stroke patients [34, 35]

A systematic review investigated the safety, applicability, and their effects on Parkinson's Disease (PD) [36]. A commercial device was used for participants in their own home, without professional supervision; besides, the authors informed about the risk of imbalance and fall (showing the importance of health professional follow-up). Regarding applicability, the selection of games and difficulty levels should be considered, in order to provide a positive feedback to patients, who might maintain motivation and adherence to exergame. In addition, overall patients enjoy performing these activities. Also, according to this review [37], exergames are able to improve clinical parameters of balance, especially if combined with traditional training for this particular physical capacity. In addition, functional clinical parameters of patients with PD seem to improve, for example, as assessed with Timed Up and Go test (gait and mobility) and Sit to Stand (muscular endurance). Thus, the modality may be effective in the symptomatic improvement of the patient with PD [36]. In complement, an intervention using 7 different Nintendo games for 12 weeks, with two sessions of exercise per week, being 30 minutes each one, the patients were stimulated to control the stability using neuromuscular strategies and mechanisms of anticipatory postural control. The results showed a potential benefit in balance and fatigue, which are influenced by multiple factors, such as motor symptoms. Therefore, such findings show the applicability of exergames as an additional tool in the treatment of PD [37].

Individuals with different neurological conditions, who may have different disabilities, through exergames may engage in physical activities with intensity regulation, facilitated by the playful and enjoyable character and the break in the transport barrier, once the consoles are portable. In a review of 10 different studies involving pathologies such as spina bifida, cerebral palsy, stroke, and spinal cord injury, the exergames ability to promote exercise at moderate intensity for these groups was shown, combining to positive psychological factors of exercise such as fun, motivation, easy learning, and physical challenge [38]. intensity and energy expenditure, make possible social interaction and psychological effects, as immersion that could lead to better enjoyment of the activity [48].

## 3 VRE FOR CHILDREN WITH CEREBRAL PALSY

Cerebral Palsy (CP) is the most frequent condition treated by rehabilitators of the infant neurological area and is the most common cause of the motor disability in children and adolescents in the world [39, 40]. In the last 40 years, the prevalence of CP has increased with 2.08/ 1000 live births (95% CI 2.02 to 2.14) [41]. In developed countries, the associated risk factors for CP are premature birth, with underweight infants and the increase of infant survivors with low weight when born [42]. Frequently, children with CP should receive physiotherapy treatment that often extends for several years or a lifetime. The repetition and above all the motivation of children during the exercises are crucial for cerebral plasticity, to improve the control of the movements and the maintenance of the posture. In line with this, virtual reality environments can elicit multisensory interactions that motivate and engage the patient in longer and more intensive sessions. The use of VR interface devices like video game consoles (low cost), positive effects have been reported in the postural control of children with CP [43, 44]. Exercise protocols with the Nintendo Wii have reduced spasticity in lower limbs and improving standing postural balance aspects that directly contribute to improve motor control and quality of life in children with CP [43, 44]. At least three mechanisms could explain such benefits: (i) repetition, (ii) sensory feedback and (iii) motivation. Each virtual game involved the successive repetition of exercises in different planes of motion in each session. In addition, each game repeats the same movement a number of times. For example, an intervention using VRE therapy caused neural activation and subsequent reorganization in the cortex of the affected side in a child with CP [45]. Therefore, repetition is the basis of neuroplasticity changes in the brain. Sensory feedback occurs by multisensory environments generated during exercise-games performance. By using an exercise protocol with balance board games, trains the postural balance in a sequence of exercises in three planes of motion: sagittal, frontal and transverse [43, 44]. The movements during games make children with CP adopt continuous weight-shifting strategies for feet, producing a mechanical stimulation to trigger the proprioceptors at this level. These strategies are orchestrated by visual feedback which has been postulated to improve balance in participants, as the video game creates the perception that they can perform more complex activities generating kinesthetic movements and consequently, proprioceptors in the lower limbs, upper limbs and the trunk are activated [46]. Such information ascends to the central nervous system (sensorimotor cortex) and descends through spinal cord performing postural adjustments necessary to the demands of each individual [47]. The motivation is achieved through various attractive and interesting exercises introduced by the consoles such as the Nintendo Wii. The possible motivation and engagement increase when using exergames may be related to different parameters, as a good balance in 

## 4 POSSIBLE NEUROBIOLOGICAL EFFECTS OF VRE

Recent advances in neuroscience have known the relationship between physical exercise and neurobiological effects which contribute to the treatment of neurodegenerative and mental diseases. According to the review of Portugal et .al [49], exercise promotes changes in blood flow, oxygenation and cerebral metabolism, synthesis, and release of neurotransmitters and neuromodulators, as well as trophic factors. These are especially related to neuroplastic mechanisms, such as neurogenesis, synaptogenesis, and angiogenesis [49]. In addition, exercise is able to reduce inflammatory cytokines and increase the synthesis of anti-inflammatory cytokines, which decrease the risk of chronic degenerative diseases [50].

**Fig. (1):**
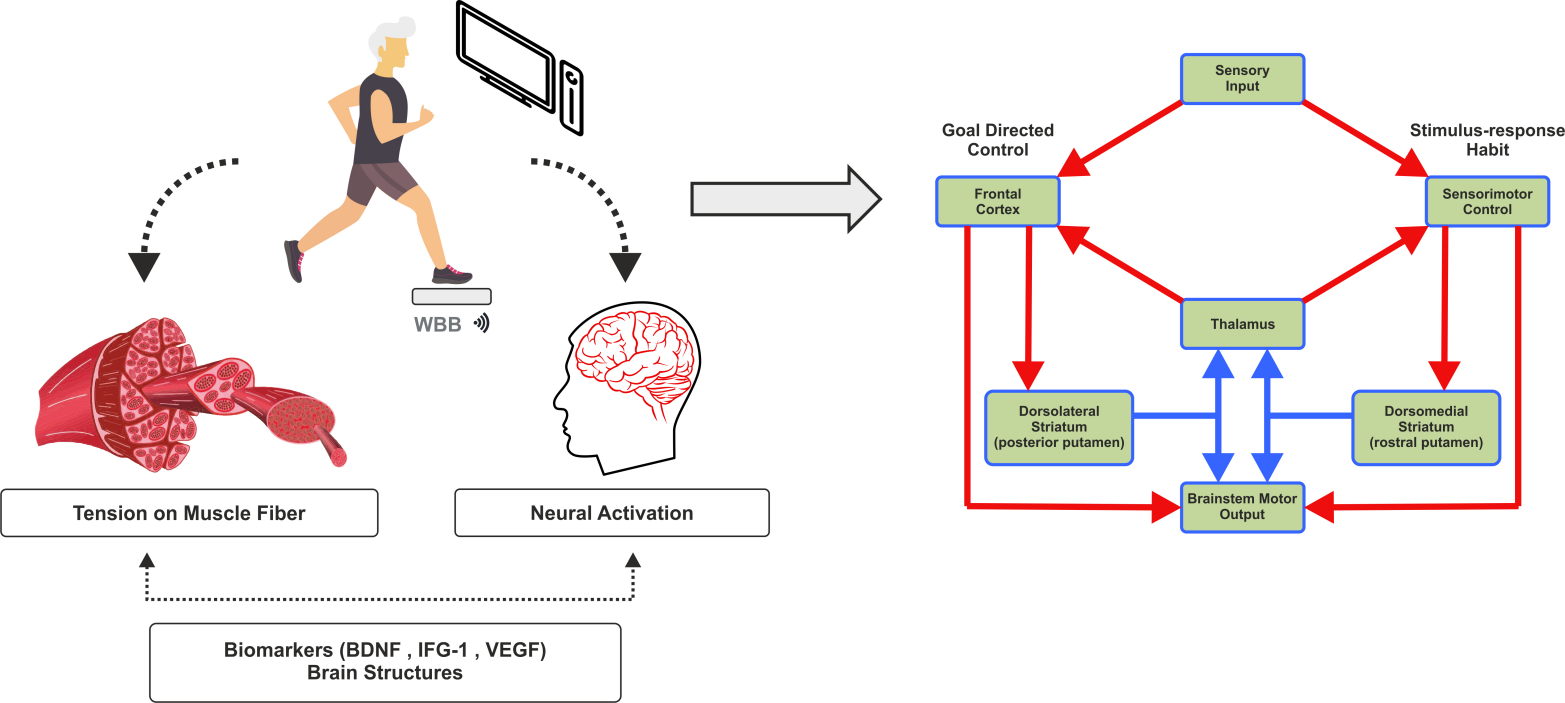
Effects of VRE on muscle and brain circuitry. Tension on muscle stimulates synthesis of trophic factors, which traffics to brain structures (hippocampus and cortex). Navigational and motor circuitry activation are potentiated by trophic factors, which may trigger neuroplasticity.

According to this context, exergames possibly promote similar effects as those for traditional exercises, since they are able to generate physical effort [51]. In addition, VRE is a dual-task, requiring not only physical but cognitive work simultaneously. During interaction with the virtual environment, the participant needs to evoke working memory, planning activities, activates inhibitory control, and makes a decision. Greater intensity of cognitive and sensorial flow may be associated with functional and structural brain adaptive modifications, also mediated by trophic factors, as brain-derived neurotrophic factor (BDNF), Glial-derived Neurotrophic Factor (GDNF), insulin-like growth factor-1 (IGF-1), Fibroblast Growth Factor-2 (FGF-2), and Vascular Endothelial Growth Factor (VEGF), which are related to neuroplasticity (neuro-genesis, synaptogenesis, and angiogenesis) [46] (Fig. **1**).

In summary, according to the physical effort provided by VRE, increased muscle requirement would elevate the synthesis of peripherally trophic factors and anti-inflammatory cytokines, which would traffic to the brain and increase neuroplastic potential in the hippocampus, frontal and parietal cortex, related to many cognitive abilities as executive function and memory, in addition to a reduction of neuroinflammation [52]. Moreover, cognitive stimulation promoted by interaction with the virtual environment would increase the efficiency of cognitive processing circuitry, especially executive functions, and could be potentiated by peripheral factors directed to the brain [52, 53]. Furthermore, we explored in the article some other hypothesis that could explain the positive effects of exergames through interaction with the digital environment, as sensory neurofeedback and activation of the mirror neuronal system. This first mechanism would work through continuous adaptation, through weight-shifting from heel to toes, stimulating proprioceptors from trunk and limbs multiple times. The information would ascend and descend in the spinal cord, provoking adaptation in the central nervous system capable of improving balance, for example [14]. For the mirror neurons system hypothesis, the interaction with an avatar on the screen would promote activation of mirror neurons, believed to be part of motor system fired when moving and observing movements, relating the observed to the action made, which could promote motor learning [13]. Therefore, in addition to the already known effects of physical exercise, exergames would increase the potential brain adaptation, the phenomenon known as neuroplasticity, which would result in improved problem-solving ability, as well as greater sensorimotor integration.

## CONCLUSION

If exercise is considered as “remedy” for its preventive potential and for the treatment of chronic diseases, it is possible to infer that an association with virtual reality could potentiate its therapeutic effects, especially in certain cases, for example, in the improvement of the cognitive functions of older adults, in augmenting balance and motor function of PD and stroke patients, as well as reducing spasticity in children with cerebral palsy. Therefore, VRE is also a “remedy.”

## References

[1] http://apps.who.int/iris/bitstream/10665/44399/1/9789241599979_eng.pdf.

[2] Jalayondeja C., Jalayondeja W., Mekhora K., Bhuanantanondh P., Dusadi-Isariyavong A., Upiriyasakul R. (2017). Break in sedentary behavior reduces the risk of noncommunicable diseases and cardiometabolic risk factors among workers in a petroleum company.. Int. J. Environ. Res. Public Health.

[3] Owen N., Sparling P.B., Healy G.N., Dunstan D.W., Matthews C.E. (2010). Sedentary behavior: Emerging evidence for a new health risk.. Mayo Clin. Proc..

[4] https://health.gov/paguidelines/pdf/paguide.pdf.

[5] Wang X., Strizich G., Hua S., Sotres-Alvarez D., Buelna C., Gallo L.C., Gellman M.D., Mossavar-Rahmani Y., O’Brien M.J., Stoutenberg M., Wang T., Avilés-Santa M.L., Kaplan R.C., Qi Q. (2017). Objectively measured sedentary time and cardiovascular risk factor control in us hispanics/latinos with diabetes mellitus: Results from the hispanic community health study/study of Latinos (HCHS/SOL).. J. Am. Heart Assoc..

[6] Lobelo F., Stoutenberg M., Hutber A. (2014). The exercise is medicine global health initiative: A 2014 update.. Br. J. Sports Med..

[7] Kohl H.W., Craig C.L., Lambert E.V., Inoue S., Alkandari J.R., Leetongin G., Kahlmeier S. (2012). The pandemic of physical inactivity: Global action for public health.. Lancet.

[8] Hardcastle S.J., Hancox J., Hattar A., Maxwell-Smith C., Thøgersen-Ntoumani C., Hagger M.S. (2015). Motivating the unmotivated: How can health behavior be changed in those unwilling to change?. Front. Psychol..

[9] Rhodes R.E., de Bruijn G.J. (2013). How big is the physical activity intention-behaviour gap? A meta-analysis using the action control framework.. Br. J. Health Psychol..

[10] Oh Y., Yang S. (2010). Defining exergames & exergaming.. Meaningful play 2010 conference proceedings http:// meaningfulplay.msu.edu/ proceedings2010/.

[11] Golubic R., Martin K.R., Ekelund U., Hardy R., Kuh D., Wareham N., Cooper R., Brage S. (2014). Levels of physical activity among a nationally representative sample of people in early old age: Results of objective and self-reported assessments.. Int. J. Behav. Nutr. Phys. Act..

[12] Meekes W., Stanmore E.K. (2017). Motivational determinants of exergame participation for older people in assisted living facilities: Mixed-methods study.. J. Med. Internet Res..

[13] Gatica-Rojas V., Velásquez R.C., Muñoz E.G. (2017). Effectiveness of a Nintendo Wii balance board exercise programme on standing balance of children with cerebral palsy: A randomised clinical trial protocol..

[14] Kommalapati R., Michmizos K.P. (2016). Virtual reality for pediatric neuro-rehabilitation: Adaptive visual feedback of movement to engage the mirror neuron system.. Conf. Proc. IEEE Eng. Med. Biol. Soc..

[15] Street T.D., Lacey S.J., Langdon R.R. (2017). Gaming your way to health: A systematic review of exergaming programs to increase health and exercise behaviors in adults.. Games Health J..

[16] Tarakci D, Ozdincler AR, Tarakci E (2013). Wii-based balance therapy to improve balance function of children with cerebral palsy: A pilot study. J. Phys. Ther. Sci..

[17] van Diest M., Stegenga J., Wörtche H.J., Verkerke G.J., Postema K., Lamoth C.J.C. (2016). Exergames for unsupervised balance training at home: A pilot study in healthy older adults.. Gait Posture.

[18] Smits-Engelsman B.C., Jelsma L.D., Ferguson G.D. (2016). The effect of exergames on functional strength, anaerobic fitness, balance and agility in children with and without motor coordination difficulties living in low-income communities.. Hum. Mov. Sci..

[19] Rosa G. M. M. V. (2012). Efeito da realidade virtual na recuperação da função motora do membro superior em paciente com AVC crônico.. Fisioterapia Brasil, Set.-Out..

[20] Taddei F., Bultrini A., Spinelli D., Di Russo F. (2012). Neural correlates of attentional and executive processing in middle-age fencers.. Med. Sci. Sports Exerc..

[21] Booth V, Masud T, Connell L, Bath-Hextall F (2014 May). The effectiveness of virtual reality interventions in improving balance in adults with impaired balance compared with standard or no treatment: A systematic review and meta-analysis.. Clin Rehabil..

[22] Maillot P., Perrot A., Hartley A. (2012). Effects of interactive physical-activity video-game training on physical and cognitive function in older adults.. Psychol. Aging.

[23] You S.H., Jang S.H., Kim Y.H., Hallett M., Ahn S.H., Kwon Y.H., Kim J.H., Lee M.Y. (2005). Virtual reality-induced cortical reorganization and associated locomotor recovery in chronic stroke: An experimenter-blind randomized study.. Stroke.

[24] de Oliveira J.M., Fernandes R.C., Pinto C.S., Pinheiro P.R., Ribeiro S., de Albuquerque V.H. (2016). Novel virtual environment for alternative treatment of children with cerebral palsy.. Comput. Intell. Neurosci..

[25] Chen Y.P., Kang L.J., Chuang T.Y., Doong J.L., Lee S.J., Tsai M.W., Jeng S.F., Sung W.H. (2007). Use of virtual reality to improve upper-extremity control in children with cerebral palsy: A single-subject design.. Phys. Ther..

[26] Kassee C., Hunt C., Holmes M.W.R., Lloyd M. (2017). Home-based Nintendo Wii training to improve upper-limb function in children ages 7 to 12 with spastic hemiplegic cerebral palsy.. J. Pediatr. Rehabil. Med..

[27] Monteiro-Junior R.S., da Silva Figueiredo L.F., Maciel-Pinheiro P.T., Abud E.L.R., Braga A.E.M.M., Barca M.L., Engedal K., Nascimento O.J.M., Deslandes A.C., Laks J. (2017). Acute effects of exergames on cognitive function of institutionalized older persons: A single-blinded, randomized and controlled pilot study.. Aging Clin. Exp. Res..

[28] Monteiro-Junior R.S., de Souza C.P., Lattari E., Rocha N.B., Mura G., Machado S., da Silva E.B. (2015). Wii-Workouts on chronic pain, physical capabilities and mood of older women. A randomized controlled double blind trial.. CNS Neurol. Disord. Drug Targets.

[29] Kappen D.L., Mirza-Babaei P., Nacke L.E. (2018). Older adults’ physical activity and exergames: A systematic review.. Int. J. Hum. Comput. Interact..

[30] Stanmore E., Stubbs B., Vancampfort D., de Bruin E.D., Firth J. (2017). The effect of active video games on cognitive functioning in clinical and non-clinical populations: A meta-analysis of randomized controlled trials.. Neurosci. Biobehav. Rev..

[31] Hung J.W., Chou C.X., Chang H.F., Wu W.C., Hsieh Y.W., Chen P.C., Yu M.Y., Chang C.C., Lin J.R. (2017). Cognitive effects of weight-shifting controlled exergames in patients with chronic stroke: A pilot randomized comparison trial.. Eur. J. Phys. Rehabil. Med..

[32] Hurkmans H.L., Ribbers G.M., Streur-Kranenburg M.F., Stam H.J., van den Berg-Emons R.J. (2011). Energy expenditure in chronic stroke patients playing Wii Sports: A pilot study.. J. Neuroeng. Rehabil..

[33] Nelson ME, Rejeski WJ, Blair SN, Duncan PW, Juiz JO, King AC, Macera CA, Castaneda-Sceppa C (2007). American recommendation of sports medicine and the american heart association.. Med Sci Sports Exerc..

[34] Saposnik G., Teasell R., Mamdani M., Hall J., McIlroy W., Cheung D., Thorpe K.E., Cohen L.G., Bayley M. (2010). Effectiveness of virtual reality using Wii gaming technology in stroke rehabilitation: A pilot randomized clinical trial and proof of principle.. Stroke.

[35] Laver KE, George S, Thomas S, Deutsch JE, Crotty M (2015 Feb 12). Virtual reality for stroke rehabilitation.. Cochrane Database Syst Rev..

[36] Barry G., Galna B., Rochester L. (2014). The role of exergaming in Parkinson’s disease rehabilitation: A systematic review of the evidence.. J. Neuroeng. Rehabil..

[37] Ribas C.G., Alves da Silva L., Corrêa M.R., Teive H.G., Valderramas S. (2017). Effectiveness of exergaming in improving functional balance, fatigue and quality of life in Parkinson’s disease: A pilot randomized controlled trial.. Parkinsonism Relat. Disord..

[38] Mat Rosly M., Mat Rosly H., Davis Oam G.M., Husain R., Hasnan N. (2017). Exergaming for individuals with neurological disability: A systematic review.. Disabil. Rehabil..

[39] Cans C. (2000). Surveillance of cerebral palsy in Europe: A collaboration of cerebral palsy surveys and registers. Surveillance of Cerebral Palsy in Europe (SCPE).. Dev. Med. Child Neurol..

[40] Tedroff K., Knutson L.M., Soderberg G.L. (2006). Synergistic muscle activation during maximum voluntary contractions in children with and without spastic cerebral palsy.. Dev. Med. Child Neurol..

[41] Johnson A. (2002). Prevalence and characteristics of children with cerebral palsy in Europe.. Dev. Med. Child Neurol..

[42] Hagberg B., Hagberg G., Beckung E., Uvebrant P. (2001). Changing panorama of cerebral palsy in Sweden. VIII. Prevalence and origin in the birth year period 1991-94.. Acta Paediatr..

[43] Gatica-Rojas V., Méndez-Rebolledo G., Guzman-Muñoz E., Soto-Poblete A., Cartes-Velásquez R., Elgueta-Cancino E., Cofré Lizama L.E. (2017). Does Nintendo Wii Balance Board improve standing balance? A randomized controlled trial in children with cerebral palsy.. Eur. J. Phys. Rehabil. Med..

[44] Gatica-Rojas V., Cartes-Velásquez R., Méndez-Rebolledo G., Guzman-Muñoz E., Lizama L.E.C. (2017). Effects of a Nintendo Wii exercise program on spasticity and static standing balance in spastic cerebral palsy.. Dev. Neurorehabil..

[45] You S.H., Jang S.H., Kim Y.H., Kwon Y.H., Barrow I., Hallett M. (2005). Cortical reorganization induced by virtual reality therapy in a child with hemiparetic cerebral palsy.. Dev. Med. Child Neurol..

[46] Kwok B.C., Mamun K., Chandran M., Wong C.H. (2011). Evaluation of the Frails’ Fall Efficacy by Comparing Treatments (EFFECT) on reducing fall and fear of fall in moderately frail older adults: study protocol for a randomised control trial.. Trials.

[47] Gatica R. (2013). Sistemas de control del movimiento humano..

[48] Lee S., Kim W., Park T., Peng W. (2017). The psychological effects of playing exergames: A systematic review.. Cyberpsychol. Behav. Soc. Netw..

[49] Matta Mello Portugal E., Cevada T., Sobral Monteiro-Junior R., Teixeira Guimarães T., da Cruz Rubini E., Lattari E., Blois C., Camaz Deslandes A. (2013). Neuroscience of exercise: From neurobiology mechanisms to mental health.. Neuropsychobiology.

[50] Walsh N.P., Gleeson M., Shephard R.J., Gleeson M., Woods J.A., Bishop N.C., Fleshner M., Green C., Pedersen B.K., Hoffman-Goetz L., Rogers C.J., Northoff H., Abbasi A., Simon P. (2011). Position statement. Part one: Immune function and exercise.. Exerc. Immunol. Rev..

[51] Peng W., Lin J.H., Crouse J. (2011). Is playing exergames really exercising? A meta-analysis of energy expenditure in active video games.. Cyberpsychol. Behav. Soc. Netw..

[52] Monteiro-Junior R.S., Vaghetti C.A., Nascimento O.J., Laks J., Deslandes A.C. (2016). Exergames: Neuroplastic hypothesis about cognitive improvement and biological effects on physical function of institutionalized older persons.. Neural Regen. Res..

[53] Anderson-Hanley C., Arciero P.J., Brickman A.M., Nimon J.P., Okuma N., Westen S.C., Merz M.E., Pence B.D., Woods J.A., Kramer A.F., Zimmerman E.A. (2012). Exergaming and older adult cognition: A cluster randomized clinical trial.. Am. J. Prev. Med..

